# A phase I study to evaluate the dosimetry and safety of [^89^Zr]Zr-DFO-AP-101, a new antibody-based radiopharmaceutical to detect misfolded SOD1 in amyotrophic lateral sclerosis

**DOI:** 10.1007/s00259-026-07853-y

**Published:** 2026-03-24

**Authors:** Etienne Croteau, Etienne Rousseau, Sébastien Tremblay, Jean-François Rousseau, Esteban Espinosa-Betancourt, Eric Lavallée, Stéphanie Dubreuil, Samia Ait-Mohand, Émilie Lareau-Trudel, Sylvie Gosselin, Virginie Carrier, Sarah Côté-Bigras, Catherine Allard, Marcel Maier, Michael Salzmann, Amélie Tétu, Marie-Pier Houde, Éric Turcotte, Brigitte Guérin

**Affiliations:** 1 Sherbrooke Molecular Imaging Centre (CIMS), Centre de Recherche du Centre Hospitalier Universitaire de Sherbrooke, 3001, 12e Avenue Nord, Sherbrooke, QC J1H 5N4 Canada; 2 Department of Medical Imaging and Radiation Sciences, Université de Sherbrooke, Sherbrooke, QC Canada; 3 Institut de Recherche sur le Cancer de l’Université de Sherbrooke (IRCUS), Université de Sherbrooke, Sherbrooke, QC Canada; 4 Neurology Service, Department of Medicine, Université de Sherbrooke, Sherbrooke, QC Canada; 5 Unité de Recherche Clinique et Épidémiologique (URCE), Centre de Recherche du Centre Hospitalier Universitaire de Sherbrooke, Sherbrooke, QC Canada; 6 Plateau d’Expertise Clinique (PEC), Centre de Recherche du Centre Hospitalier Universitaire de Sherbrooke, Sherbrooke, QC Canada; 7 AL-S Pharma AG, Schlieren, Zurich Switzerland

**Keywords:** Dosimetry, PET imaging, Amyotrophic lateral sclerosis (ALS), Misfolded superoxide dismutase-1, [^89^Zr]Zr-DFO-AP-101

## Abstract

**Purpose:**

Misfolded superoxide dismutase-1 (mSOD1) is an abnormal protein observed in amyotrophic lateral sclerosis (ALS) and constitutes a therapeutic target. The present study evaluated the biodistribution, dosimetry, and safety of a new antibody-based radiopharmaceutical, [^89^Zr]Zr-DFO-AP-101, targeting mSOD1.

**Methods:**

Seven control participants and one patient with ALS received 41 ± 3 MBq of [^89^Zr]Zr-DFO-AP-101. They were followed up with five whole-body positron emission tomography (PET) scans over 10 days. Semi-automatic segmentation was performed on the images to derive time-activity curves, radiotracer effective half-life and dose exposure.

**Results:**

Total elimination of the radiotracer (urinary and hepatobiliary) was 25–30% after three days and reached a plateau after a week. At 2 h post-injection, ~ 60% of the radiopharmaceutical remained in the blood pool, with a biological half-life of 53 h. The liver was the dose-limiting organ with 0.84 mSv/MBq in males, 1.07 mSv/MBq in females, and 1.23 mSv/MBq in the female ALS patient. The spleen, adrenal glands, kidney, and heart wall were the other most irradiated organs. Average effective doses were 0.21 mSv/MBq for males, 0.28 mSv/MBq for females, and 0.31 mSv/MBq for the female ALS patient. Tracer uptake in the spinal cord and vertebrae of the ALS patient, on Days 7 and 10, was more than one standard deviation higher than for the control female participants. No serious adverse event was observed.

**Conclusions:**

The single dose of [^89^Zr]Zr-DFO-AP-101 was safe for all participants. It provided good image quality for the biodistribution and dosimetry analysis over 10 days. Further studies are needed to demonstrate the efficacy of ALS diagnosis through PET imaging. https://www.clinicaltrials.gov/study/NCT05974579.

**Supplementary Information:**

The online version contains supplementary material available at 10.1007/s00259-026-07853-y.

## Introduction

Amyotrophic lateral sclerosis (ALS), also known as Lou Gehrig’s Disease, is a progressive and lethal neurodegenerative disease affecting approximately 7.7 in 100,000 people in the United States, with a higher prevalence among males, Caucasians and non-Hispanics, and an age of onset typically between 60 and 69 years of age [1]. Supporting the geographic variability in ALS epidemiology, a meta-analysis of 44 studies across 45 geographic regions reported incidence rates ranging from 0.5 per 100,000 person-years in Asia to 2.4 per 100,000 in Western Europe [2]. Respiratory failure is the leading cause of death in ALS, and some patients require permanent assisted ventilation as the disease progresses [3].

Hereditary forms of ALS account for 5–10% of cases [1], with 12–20% of these linked to mutations in the superoxide dismutase 1 (SOD1) gene [4]. These mutations lead to misfolding and aggregation of the SOD1 protein, ultimately resulting in motor neuron degeneration [5–8]. However, wild-type SOD1 was also associated with misfolding in sporadic ALS, suggesting a role in this pathogenesis even in the absence of mutations [5, 9–11]. This hypothesis is also supported by preclinical trials on transgenic mice that mimic the ALS pathology with misfolded wild-type, non-mutated, SOD1 [12, 13]. Currently, there is a lack of diagnostic tools for ALS and its subtypes, resulting in misdiagnosis or delayed diagnosis and significant disease progression before initiating therapy [14].

Biomarkers that can facilitate ALS diagnosis, aid in prognosis, and measure drug pharmacodynamics are needed to accelerate therapeutic development for patients with ALS. Positron emission tomography (PET) imaging with an appropriate radiopharmaceutical shows great potential as a biomarker for ALS, given that it would permit visualization of the central nervous system pathology in individuals living with the disease. PET imaging, known for its sensitivity and selectivity, was used with different radiotracers to explore the pathophysiology of ALS. Among these, [^18^F]FDG was the most widely used to assess glucose metabolism across various brain regions. In ALS, glucose metabolism can show both reduced and increased activity in different brain areas [15]. Various neuroinflammation-targeted radiotracers showed increased uptake, suggesting the presence of neuroinflammation in ALS [15]. Most studies using neuronal density PET radiotracers have shown a reduction in binding, which is a characteristic of the motor neuron degeneration observed in ALS [15]. However, there is a gap in research focused on imaging the aggregation of toxic proteins, including those linked to SOD1 and C9ORF72 mutations [15].

AP-101 is a fully human monoclonal antibody with selective binding to misfolded superoxide dismutase-1 (mSOD1). This antibody has been shown to identify mSOD1 in autopsied spinal cord tissue of both familial ALS (fALS) and sporadic ALS (sALS) patients regardless of their SOD1 genotype [13]. A multicenter phase 2 study was conducted to evaluate the safety, tolerability, pharmacodynamic markers, and pharmacokinetics of AP-101 in patients with fALS and sALS (NCT05039099). This randomized, double-blind, placebo-controlled trial enrolled 52 patients with sALS and 21 with fALS, who completed a 24-week treatment period followed by a 24-week open-label extension and a safety follow-up phase. Although detailed results have not yet been released, AL-S Pharma AG reported that AP-101 demonstrated a favourable safety profile and produced clinically meaningful improvements in outcome measures, as well as stabilization of key biomarkers. Therefore, it is a good candidate for in vivo assessment of mSOD1 via PET imaging if labelled with a suitable radioisotope.

Our team has developed and validated, in a mouse model, the new radiopharmaceutical [^89^Zr]Zr-DFO-AP-101 targeting mSOD1 [16]. In the preclinical phase, we have shown that [^89^Zr]Zr-DFO-AP-101 was stable in vitro and in plasma, that the non-radioactive Zr-DFO-conjugate retained its affinity and specificity for mSOD1, and that [^89^Zr]Zr-DFO-AP-101 displayed a specific uptake into the spinal cord, lumbar vertebrae and femoral head of the murine ALS model [16]. The detection of mSOD1 in the vertebrae and other joints with [^89^Zr]Zr-DFO-AP-101 was corroborated by immunohistology using AP-101 and additional antibodies recognizing mSOD1 in cartilage cells across these tissues [16]. In the murine ALS model, [^89^Zr]Zr-DFO-AP-101 also localized to mSOD1 aggregates within the spinal cord, and co-injection of excess AP-101 markedly reduced both the number of detectable aggregates and the intensity of the remaining signals [16]. Together, these preclinical findings provided a strong rationale for evaluating [^89^Zr]Zr-DFO- AP-101 in ALS patients. It should be noted that the uptake of [^89^Zr]Zr-DFO-AP-101 in the brain and spinal cord of volunteers and ALS patient is expected to be low, mainly because the blood–brain and blood–spinal cord barriers are highly selective and restrict the entry of large molecules such as antibodies into the central nervous system [17].

The primary objectives of the present phase I study were to evaluate the biodistribution, dosimetry, and safety of the radiopharmaceutical [^89^Zr]Zr-DFO-AP-101 in control participants and patients with confirmed ALS via PET imaging. The secondary objective was to evaluate the pharmacokinetics of [^89^Zr]Zr-DFO-AP-101 over time. An exploratory objective was to assess the differential accumulation of mSOD1 in the spinal cord of patients affected with ALS compared to control participants.

## Materials and methods

### Preparation of desferrioxamine (DFO)-AP-101

The DFO-AP-101 antibody was prepared as previously described [16]. Briefly, AP-101 (Neurimmune, Switzerland) was first buffer-exchanged by four sequential washes with phosphate-buffered saline (PBS, trace metals basis, pH 7.2) using 50 kDa ultrafiltration units (Amicon^®^ Ultra-05), followed by a final wash with 0.1 M sodium bicarbonate (NaHCO₃, pH 8–9). The concentrated samples were pooled, adjusted to ~ 500–600 µL with NaHCO₃, and transferred to a reaction vial. Conjugation was performed by incubating AP-101 with three molar equivalents of *p*-SCN-Bz-DFO for 1 h at 37 °C, keeping dimethylsulfoxyde below 2%. The reaction mixture was purified via repeated centrifugal filtration using PBS. The final DFO-AP-101 conjugate was pooled and stored at 4 °C. Characterization was performed by size-exclusion high performance liquid chromatography (HPLC), and the average DFO-to-AP-101 ratio was determined by isotopic dilution [16, 18]. The DFO-AP-101 concentration was measured using a bicinchoninic acid assay [16, 19].

### Preparation of [^89^Zr]Zr-DFO-AP-101

[^89^Zr]Zr-DFO-AP-101 was prepared following Good Manufacturing Practices (GMP), using an automated cassette-based module [16] and sterile filtration in a clean room. The radiolabeling was performed by incubating 3 mg of DFO-AP-101 with 717–947 MBq of [^89^Zr]ZrCl_4_ for 40 min at 37 °C. The specific activity was estimated at the end of incubation based on the radiolabeling yields, the amount of activity incubated, and the quantity of DFO-AP-101 used. [^89^Zr]Zr-DFO-AP-101, formulated in a 10 mL solution constituted of saline (0.9%) and gentisic acid (10 mg), was released for clinical use after prerelease quality control specifications were met. Details regarding eligible volunteers, quality control tests and biodistribution data can be found in the supplementary material and methods section (Supplemental Table 1).

### Study design and participant selection

This phase I, single-center, open-label study was conducted at the Sherbrooke Molecular Imaging Center (CIMS) in Canada. The study was approved by the institutional research ethics board, and all participants provided written informed consent prior to any procedure. The investigational use of [^89^Zr]Zr-DFO-AP-101 was approved by Health Canada, and the study was registered on *Clinicaltrials.gov* (*NCT05974579*).

Control participants without neurologic conditions, aged 50 years or older, and patients diagnosed with ALS based on the El Escorial criteria [20], aged 18 or older, were recruited in a convenience sample. Participants with significant hepatic disorders, severe chronic kidney disease, severe active psychiatric illness, or other progressive health conditions were excluded. Additionally, patients with ALS were excluded if they had a tracheostomy or required positive pressure ventilation more than 4 h/day while awake to treat their symptoms. All eligible participants were subsequently invited to undergo physical exams, electrocardiogram test, vital signs measurements, blood samples, and PET imaging at Day 0 (radiopharmaceutical administration), 1, 3, 7, and 10 (± 1 day).

### Safety assessment and blood analysis

A safety follow-up was performed for 14 days after the injection. This included physical examinations by neurologists or qualified investigators at the first visit, vital sign measurements, blood cell count, and chemistry panels, as well as electrocardiograms performed on each PET scan day. Urine and blood samples were also collected to compare the blood activity with the imaging dataset. Urine and plasma stability study was realized from blood sample and urine as previously described [20, 21]. Moreover, concomitant medications and adverse events were recorded at each visit, and the qualified investigators assessed their clinical relevance.

### [^89^Zr]Zr-DFO-AP-101 administration and PET/Computed Tomography (CT) imaging

[^89^Zr]Zr-DFO-AP-101 (41 ± 3 MBq) was administered intravenously, and whole-body PET/CT were performed 2 h and 1-, 3-, 7-, and 10-days post-injection. All imaging was performed on a Biograph Vision 600 scanner (Siemens, Erlangen, Germany) with a 26 cm field of view using flow-motion acquisition. A NEMA IEC PET body phantom containing 40 MBq of ^89^Zr and a 4:1 activity ratio was used to validate the flow motion speed acquisition. The acquisition time (34 ± 3 min) was adjusted according to the height of the participant. PET/CT whole-body images were reconstructed with a matrix size of 220 × 220 and isotropic voxels of 3.3 mm^3^ using an iterative algorithm with point-spread function modelling and time-of-flight.

Low-dose CT scans, used for segmentation and attenuation correction, were acquired using an energy of 120 kV, and automated current modulation tailored to the individual patient size and shape (CARE Dose4DTM from Siemens). The Advanced Modeled Iterative Reconstruction algorithm (ADMIRETM) was used for image reconstruction with a strength of 3 and Br38 kernel. An isotropic voxel size equivalent to the PET voxel size was used. The average effective dose from the CT was 3.0 ± 0.7 mSv (head-to-toe) using a k-factor of 0.014. The CTDIvol was 1.5 ± 0.7 mGy.

### Image analysis

**Fig. 1:**
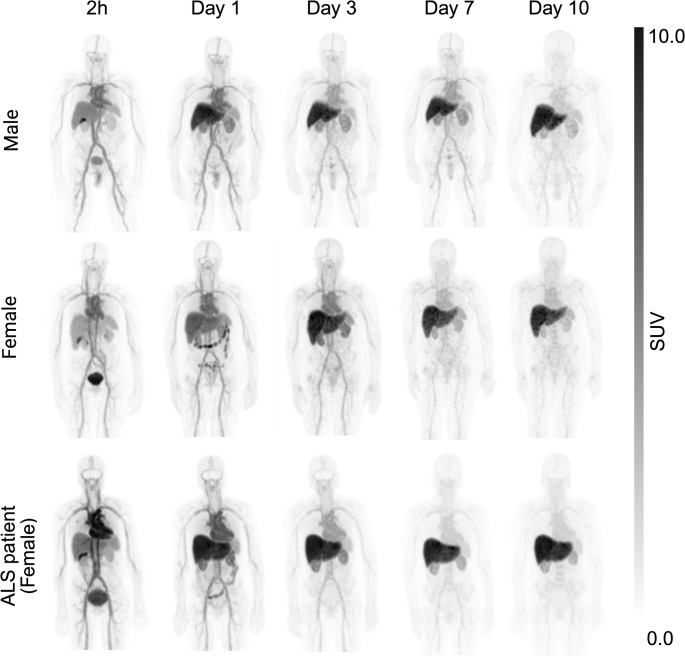
Decay-corrected whole-body maximal-intensity projection PET/CT images of healthy volunteers (male and female), and an ALS patient injected with [^89^Zr]Zr-DFO-AP-101

**Fig. 2:**
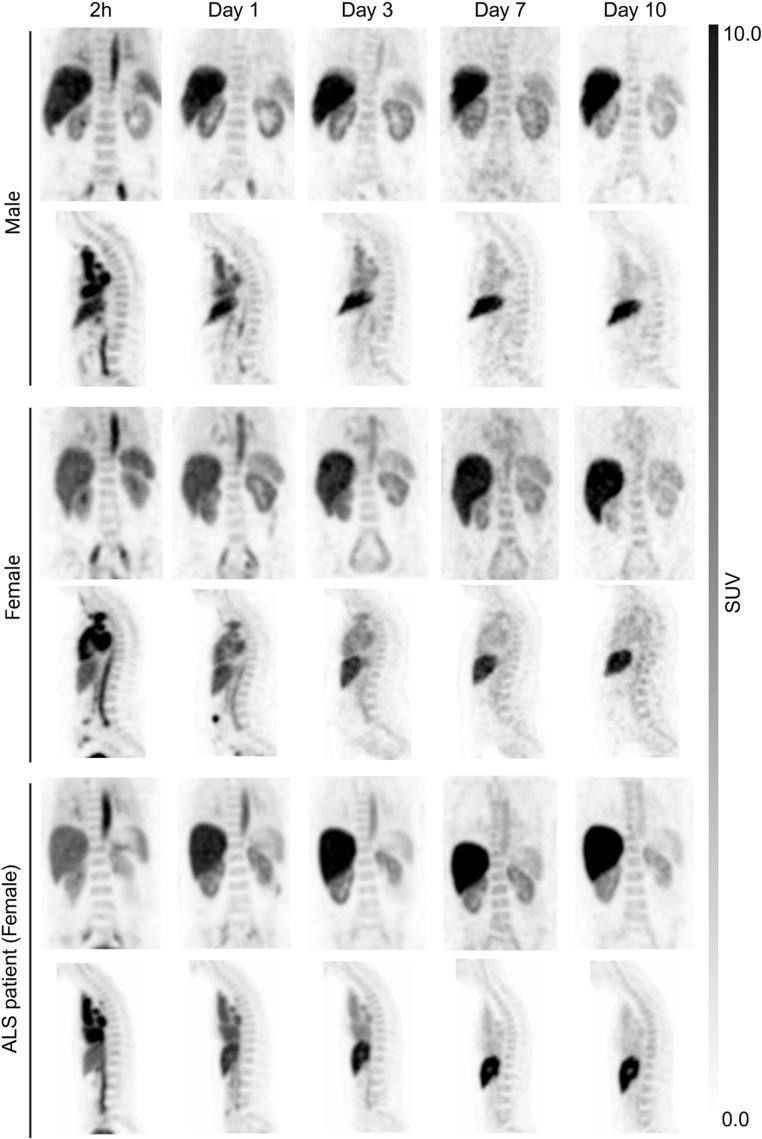
Decay-corrected PET images showing the vertebrae (coronal view) and spinal cord (sagittal view) in healthy volunteers (male and female), and in the ALS patient following injection of [^89^Zr]Zr-DFO-AP-101

Segmentation was performed on the CT whole-body images using deep learning. The images were provided to the segmentation algorithm without any preprocessing. They were segmented using the *Whole-body segmentation TS2* pretrained model (v2.0.1) from the MONAI Auto3DSeg library (https://github.com/Project-MONAI) in 3D slicer (v. 5.6.2; https://github.com/lassoan/SlicerMONAIAuto3DSeg). The results were examined visually, and manual corrections were applied as needed (Supplemental Fig. 1). The PET and CT images were registered in PMOD (v. 3.8, Bruker), and the CT segmentation was applied to the PET images (Supplemental Figs. 1–3) to assess [^89^Zr]Zr-DFO-AP-101 biodistribution and time-activity curves from target organs and regions of interest relevant to ALS. Volumes of interest (VOIs) were drawn on organs showing visible tracer uptake in the PET images. Average activity concentrations for the liver, spleen, and kidneys were determined by delineating VOIs that encompassed most of each organ at each time point. The blood activity was obtained from the left cardiac ventricle cavity and validated against the blood sample activity. No partial volume correction was applied for any region of interest. Blood volumes were estimated with a model considering gender, height, and weight [22]. Red marrow activity was derived from VOIs on the marrow uptake seen on lumbar vertebrae. Urinary bladder activity was assessed using a VOI encompassing the entire bladder, as visualized on the PET images. At each time point, activity concentrations within these VOIs were expressed as a percentage of the injected dose (%ID). Biological and effective half-lives were extracted from the time-activity curves using a monoexponential fit (GraphPad, Prism v. 9.5.1). The tissue and blood uptakes were decay-corrected and expressed as %ID. Localised mean uptake values were calculated for the spinal cord (cervical 2 to lumbar 4), thoracic (1–12) and lumbar (1–4) vertebrae, and distal femoral head. Values were decay-corrected and reported as %ID per gram (%ID/g) of tissue. The whole-body scan acquired 2-hour post-injection without voiding the bladder was considered as the total injected activity. Maximum intensity projection PET images (Figs. 1 and 2) are expressed as standardized uptake value (SUV), which involves normalizing the measured activity concentration in megabecquerel per milliliter (MBq/mL) by the injected dose and patient weight.

### Normal-tissue dosimetry

Non-decay-corrected biodistribution data (Supplemental Tables 2–4) were used to generate time-activity curves for [^89^Zr]Zr-DFO-AP-101 and dosimetry calculation. Organ time-integrated activity was calculated by analytic integration of mono- or dual-exponent fits for the organs and blood-pool time-activity (%ID) curves. The calculated residence times were then entered into the OLINDA/EXM software, version 2.2.3 (Hermes Medical Solutions) to calculate organ doses and the effective dose in mSv/MBq.

### Statistical analysis

Continuous data are presented with mean and standard deviation. Categorical variables are presented with frequencies and proportions. The analyses were performed using R, version 4.4.2, and OLINDA.

## Results

### Preparation of [^89^Zr]Zr-DFO-AP-101

The [^89^Zr]Zr-DFO-AP-101 was obtained with radiochemical yields greater than 60%. Radiochemical impurities such as ^88^Zr and ^88^Y were produced at a low level, below 2% [16] within the 29-hour after end-of-synthesis shelf life. The vial contained 3.0 mg of DFO-AP-101, with an activity of 300–500 MBq. The final volume for injection was based on the HEPES or gentisic acid contents, for which the specifications are ≤ 200 µg/injection and ≤ 5 mg/injection, respectively. [^89^Zr]Zr-DFO-AP-101 solutions for injection had a pH of 4.9–5.5, a radiochemical purity of greater than 99%, and a radionuclidic purity of at least 99.4%. The radiochemical purity was determined by HPLC immediately after purification. In addition, a stability study was performed over 29 h to confirm that the radiochemical purity was maintained up to the time of injection. The specific activity was estimated based on the radiolabeling yields, the amount of activity incubated, and the quantity of DFO-AP-101 used at the end of the incubation, resulting in an average value of 262 ± 21 MBq/mg. In all cases, injections were performed within 24 h following purification. All prepared batches complied with standard requirements for intravenous injections, including sterility and endotoxin level. A preliminary stability assessment performed in human samples showed that [^89^Zr]Zr-DFO-AP-101 remained stable in vivo, with no detectable free ^89^Zr in blood or urine at 2 h post-injection (Supplemental Fig. 4).

### Patient characteristics and safety

Eligibility was assessed for 33 volunteers, and a final number of eight participants (seven control participants and one with ALS) were included in the study and received the radiopharmaceutical (Supplemental Fig. 5). Their characteristics are reported in Table 1. The patient with ALS was diagnosed with a sporadic form in April 2023, had an ALSFRS-R score of 33 and a slow vital capacity of 82%. All controls and the ALS patient completed the study; however, 1 male participant did not undergo the Day-7 scan due to a technical failure. Between the screening visit and the last call on Day 14, 16 minor adverse events were observed among 6 of the study participants and are presented in Table 2. All blood assessments were found to be within the standard range or not clinically significant (Supplemental Table 5).

**Table 1:** Participant characteristics

Variables	Mean ± standard deviation or frequencies (%)
Age at consent (years)	59.9 ± 7.5
Biological sex	
Female	5 (62.5)
Male	3 (37.5)
Ethnicity	
Caucasian, Non-Hispanic	8 (100)
Body Mass Index	27.9 ± 3.5
Comorbidities*	
Past cancer	0
Respiratory^a^	2 (25)
Cardiometabolic^b^	2 (25)
Gastrointestinal^c^	4 (50)
Liver^d^	1 (12.5)
Urogenital^e^	4 (50)
Musculoskeletal^f^	3 (37.5)
Mental health	0
Tobacco use	
Current smoker	3 (37.5)
Non-smoker	5 (62.5)
Alcohol use	
None	2 (25)
Occasionally	6 (75)
Drug use	
None	7 (87.5)
Occasionally^g^	1 (12.5)
Net dose of injected radiopharmaceutical (MBq)^h^	40.91 ± 2.74
Acquisition time (min)^h^	33.88 ± 3.14

* Medical history from the last 5 years

^a^ rhinitis, asthma

^b^ hypertension, type I diabetes, dyslipidaemia, hyperlipidaemia

^c^ gastroesophageal reflux, haemorrhoids, gastrostomies

^d^ steatosis

^e^ endometriosis, herpes, urinary retention, inguinal hernia

^f^ chronic pain, arthrosis, hip replacement surgery

^g^ cannabis

^h^ at Day 0, the day of injection and first imaging

**Table 2:** Adverse events throughout the study

Participant	Description of AE	Time between injection of radiopharmaceutical and onset of AE (days)	Treatment required	Severity	Causality
001	Headache during 1 h	4	No	mild	NR
002	Fatigue during 1 h	0	No	mild	PR
	Hematoma, right arm (at the site of blood draw) for 11 days	1	No	mild	NR
003	Fatigue for 15 days	0	No	mild	PR
	Headache for 1 day	2	Yes, acetaminophen	severe	PR
	Haemorrhoid flare-up for 2 days	5	Yes	severe	NR
	Recurrence of genital herpes (itching) for one day	6	Yes, famciclovir	severe	NR
	Common cold, at least 7 days	12	Yes, guaifénésine, pseudoephedrine, acetaminophen	severe	NR
	Conjunctivitis, both eyes	12	Yes, polymyxin B, gramicidin	severe	NR
006	Mild fatigue for one day	3	No	mild	NR
	Mouth dryness for two weeks	3	No	mild	NR
	Increased discharge from the stoma for 7 days	3	No	mild	NR
	Bruise left arm (at the site of blood draw) for 9 days	3	No	mild	NR
	Bruise right arm (at the site of injection) for 9 days	3	No	mild	PR
011	Skin rash on left upper limb (close to the site of injection) for 3 h	0	No	mild	NR
012	Night sweats for two nights after the injection	1	No	mild	NR

*AE* Adverse events, *NR* Not related, *PR* Possibly related

### PET/CT imaging and biodistribution

**Fig. 3:**
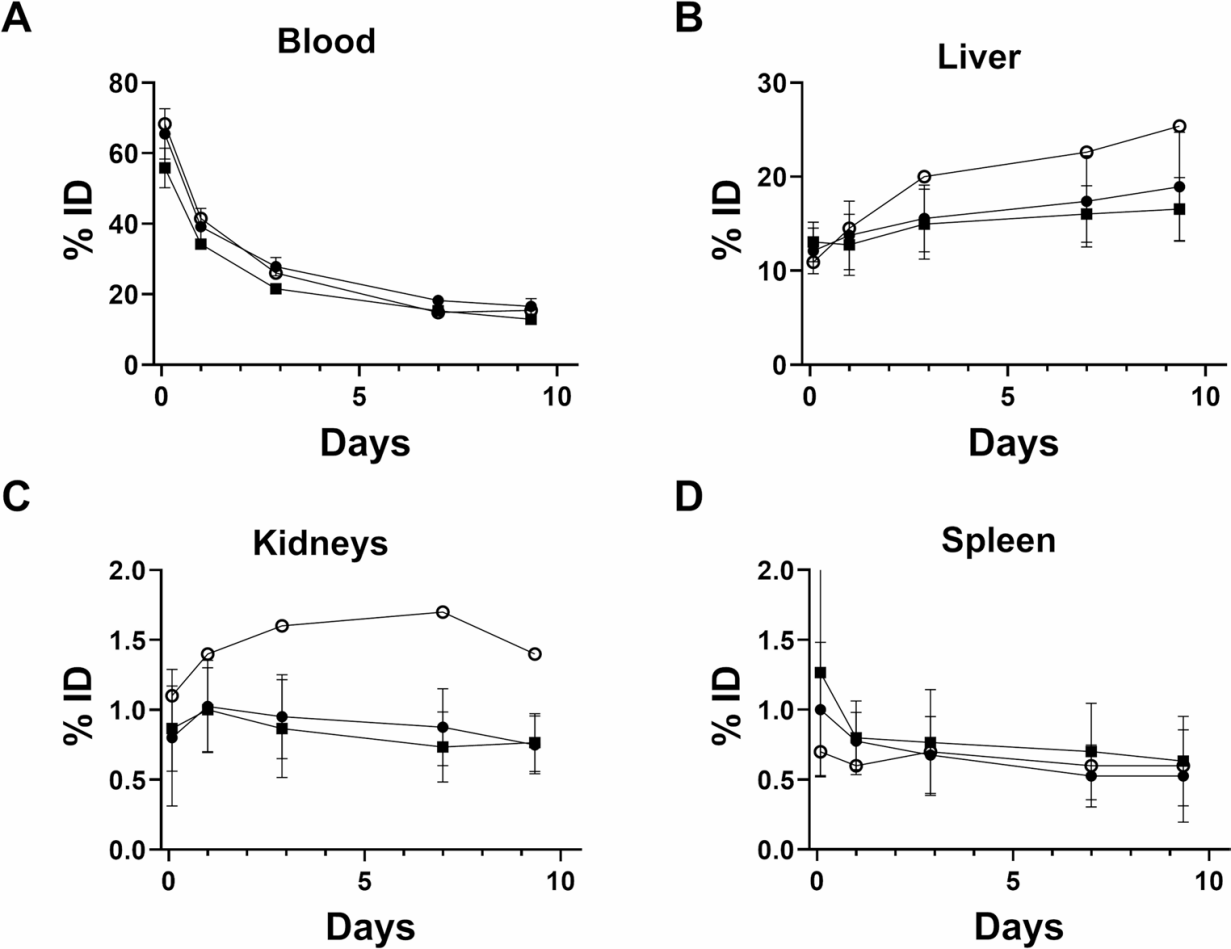
Decay-corrected time-activity (%ID) curves of [^89^Zr]Zr -DFO-AP-101 in the blood (**A**), liver (**B**), kidneys (**C**), and spleen (**D**) for male participants (black square), female participants (black circle), and a female ALS patient (white circle)

**Fig. 4:**
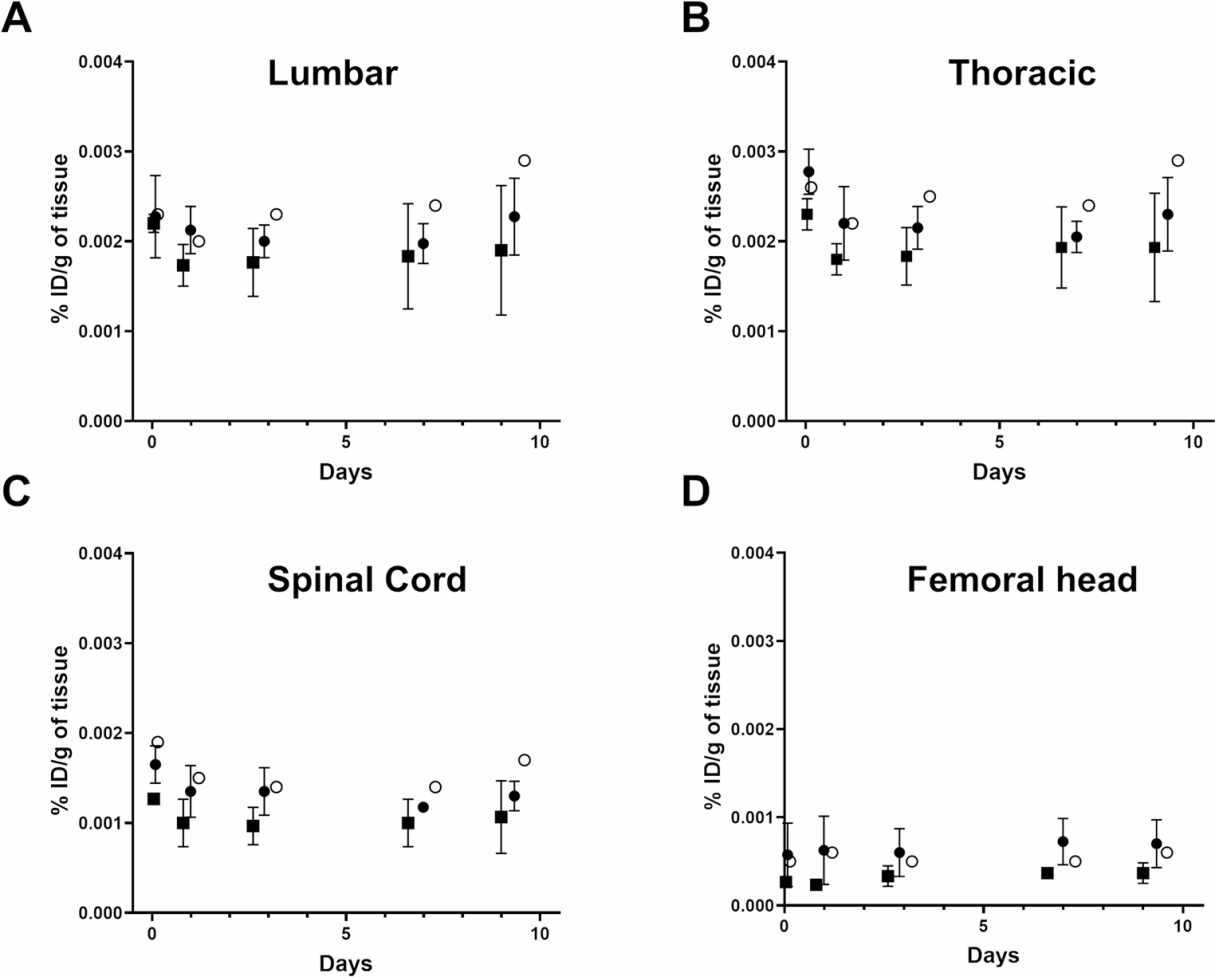
Decay-corrected time-activity graphs (%ID/g) of [^89^Zr]Zr -DFO-AP-101 in the lumbar (**A**) and thoracic (**B**) vertebrae, as well as the spinal cord (**C**) and the femoral head (**D**) for male participants (black square), female participants (black circle), and a female ALS patient (white circle)

Whole-body PET/CT imaging of 7 healthy volunteers and the ALS patient revealed that the ^89^Zr radioactivity after intravenous injection of [^89^Zr]Zr-DFO-AP-101 first underwent early elimination by the kidneys to the urinary bladder, followed by prolonged elimination by the liver (Figs. 1 and 2). As with other antibody-based PET radiotracers, the optimal imaging window was 5 to 7 days post-administration, when blood activity was low and the signal/background ratio was at a maximum. The effective half-life of the radiopharmaceutical was estimated at 53 h. Of all the organs, the liver had the highest uptake values at all time points (Supplemental Tables 6–8). A time-dependent decline in tracer uptake was observed in most normal tissues; however, uptake increased in the liver, kidneys and remained almost stable in the vertebrae and spinal cord (Figs. 2, 3 and 4, Supplemental Tables 6–11). Furthermore, we observed that the kidney and liver uptake were higher in the ALS patient (Fig. 3).

Visualization of the spinal cord and vertebrae, the structures of interest for assessing mSOD1 presence in ALS [16], was optimal when the blood activity was below 20% and regional uptake was still increasing or had reached a plateau. This occurred approximately one week after the injection (Figs. 2, 3 and 4). One should note that the uptake in the vertebrae (lumbar and thoracic) and the spinal cord of the female ALS patient was higher compared to that of the female volunteers at days 7 and 10. It was also higher than for the male volunteers on Day 10 (Fig. 4A-C and Supplemental Tables 9–11). PET imaging analysis of the distal femoral head showed that the %ID/g of tissues in the female ALS patient remained relatively stable over time and was similar than that observed in healthy female and male volunteers at all evaluated time points (2 h, Days 1, 3, 7, and 10 post-injection, Fig. 4D, Supplemental Tables 9–11).

### Dosimetry

The pharmacokinetics of [^89^Zr]Zr-DFO-AP-101 in humans closely mirrored that of rodent studies [16]. Two hours after the administration of this radiopharmaceutical, about a third of the activity localized to organs, while the rest remained in the blood. Liver and kidney uptakes were generally greater in the ALS patient than in control participants. Absorbed dose estimates from the OLINDA/EXM software are provided in Table 3. The five organs with the highest absorbed dose were, in descending order, the liver, spleen, gallbladder wall, heart wall, and adrenal glands for males. In females, the liver received the highest dose, followed by the heart wall, spleen, adrenal glands, and kidneys. Therefore, for both sexes, the liver was the dose-limiting organ. The calculated effective doses of [^89^Zr]Zr-DFO-AP-101 were 0.21 mSv/MBq in males, 0.28 mSv/MBq in females, and 0.31 mSv/MBq in the ALS patient.

**Table 3:** Organ doses [mSv/MBq] estimate for [^89^Zr]Zr-DFO-AP-101

	Dose (mSv/MBq)		
Target Organ	Males (*n* = 3)	Females (*n* = 4)	ALS Female (*n* = 1)
Adrenals	0.39	0.49	0.53
Brain	0.07	0.10	0.09
Breasts		0.18	0.18
Esophagus	0.25	0.33	0.36
Eyes	0.11	0.14	0.13
Gallbladder Wall	0.46	0.39	0.42
Left Colon	0.21	0.27	0.28
Small Intestine	0.21	0.24	0.28
Stomach Wall	0.25	0.30	0.30
Right Colon	0.24	0.27	0.27
Rectum	0.18	0.23	0.21
Heart Wall	0.44	0.61	0.63
Kidneys	0.38	0.46	0.59
Liver	0.84	1.07	1.23
Lungs	0.27	0.32	0.46
Prostate/Ovaries	0.19	0.23	0.22
Pancreas	0.27	0.39	0.42
Salivary Glands	0.14	0.16	0.15
Red Marrow	0.20	0.24	0.28
Osteogenic Cells	0.18	0.22	0.23
Spleen	0.46	0.52	0.49
Thymus	0.22	0.28	0.31
Thyroid	0.16	0.19	0.20
Urinary Bladder Wall	0.35	0.25	0.22
Testes/Uterus	0.14	0.23	0.22
Total Body	0.16	0.22	0.23
Effective Dose	**0.21**	**0.28**	**0.31**

## Discussion

The need for reliable ALS biomarkers is critical, as diagnosis is frequently delayed by 10–16 months due to phenotypic heterogeneity and limited early recognition [16, 23]. A recent proof-of-concept study using a novel SOD1 seed-amplification assay demonstrated the ability to detect mSOD1 in spinal cord and motor cortex samples from both sALS and fALS patients, supporting its potential as a biomarker for most ALS cases [24]. However, this method is invasive, and assay conditions must be adapted for less accessible biospecimens, underscoring the urgent need for non-invasive approaches to quantify mSOD1 in ALS patients.

[^89^Zr]Zr-DFO-AP-101 is a promising novel PET tracer for the imaging of mSOD1 in ALS patients [16]. In this study, we present, for the first time, the whole-body distribution, pharmacokinetics, and radiation dosimetry of the tracer in both healthy controls and an ALS patient. Most importantly, we also established a robust and well-validated radiosynthesis protocol for producing GMP-grade [^89^Zr]Zr-DFO-AP-101. This protocol proved highly reproducible, enabling the successful production of 10 batches of [^89^Zr]Zr-DFO-AP-101 with consistently high radiochemical yield, purity, and molar activity. The activity values (262 ± 21 MBq/mg) varied only slightly between preparations, suggesting that such differences are unlikely to have a meaningful impact on the dosimetry results.

Following intravenous administration, [^89^Zr]Zr-DFO-AP-101 initially showed rapid renal elimination of a small fraction of the tracer, followed by notable hepatic accumulation—a distribution pattern commonly observed with other ^89^Zr-labeled antibodies such as anti-PD-1 [^89^Zr]Zr-pembrolizumab [25] and [^89^Zr]Zr-trastuzumab [26], likely attributable to interactions with Fc-γ receptors [16]. [^89^Zr]Zr-DFO-AP-101 desmonstrated slow blood clearance, a characteristic typical of large antibody-based radiopharmaceuticals [27, 28]. Uptake in the kidneys and urinary bladder may be partially attributable to radioactive metabolites of [^89^Zr]Zr-DFO-AP-101; however, our preliminary in vivo stability study at 2 h post-injection indicates that the tracer remains intact, with no detectable free ^89^Zr, providing confidence in its stability and specificity for further in vivo imaging studies.

Image quantification results and radiation dose estimates were generally consistent across participants, except for the ALS patient, who exhibited elevated uptake in the kidneys and liver compared to others. This increased uptake may be partly explained by reduced mobility, which is known to affect organ perfusion, tracer clearance, and systemic metabolism [29]. Limited mobility has also been associated with impaired renal function, which has been reported to increase physiological tracer uptake in the liver and blood pool in some PET studies [30–32]. In addition to these physiological factors, pathological mechanisms may also contribute. Significant levels of mSOD1 have been detected in the liver and kidneys of SOD1 transgenic mice using ELISA- and antibody-based methods [33–35], as well as in a patient with mSOD1 fALS [36]. Therefore, peripheral accumulation of mSOD1 may contribute to the higher tracer uptake observed in the liver and kidneys of the ALS patient.

The absence of a clear difference in brain uptake between healthy volunteers and the ALS patient may be attributed to several factors, including the limited sample size, disease heterogeneity, and the well-known challenges of antibody penetration across the blood–brain and blood–spinal cord barriers [17]. Additionally, it is possible that the ALS patient did not express mSOD1. Consequently, the lack of a detectable difference in these regions should not be interpreted as evidence against target relevance, but rather as a limitation of the current dataset that warrants further evaluation in a larger cohort.

The slightly increased uptake observed at later time points in the vertebrae and the spinal cord compared with healthy female participants should be interpreted with caution, given that this observation is based on a single female ALS patient. Nevertheless, this finding is consistent with trends observed in our preclinical studies [16]. Regarding skeletal uptake, we did evaluate tracer accumulation in larger bones. Notably, femoral head uptake in the ALS patient was lower than that observed in female volunteers at all measured time points, arguing against a generalized increase in bone uptake or a dominant contribution from unbound ^89^Zr. In addition, our in vivo stability data did not indicate significant release of free ^89^Zr at 2 h post-injection, further supporting that the observed vertebral signal is unlikely to be driven by nonspecific bone sequestration.

In contrast to our preclinical findings, PET imaging in the human subject revealed a different uptake pattern, with the %ID/g of tissues in the femoral head of the female ALS patient consistently lower than that observed in healthy female volunteers, whereas [^89^Zr]Zr-DFO-AP-101 showed specific and higher femoral head uptake in the murine ALS model compared with control mice. These discrepancies may reflect species-related differences in target expression, disease biology, and antibody pharmacokinetics. Moreover, the murine model represents a controlled genetic form of ALS with confirmed mSOD1 expression, while mSOD1 expression was not assessed in the human patient.

We also acknowledge that differences in %ID may be influenced by factors unrelated to ALS pathology. Such effects cannot be excluded in the present study and will be carefully addressed in future and larger-scale investigations.

Due to the prolonged circulation time of [^89^Zr]Zr-DFO-AP-101 and the long physical half-life of ^89^Zr, the resulting radiation dose of this radiopharmaceutical is higher compared to that of small-molecule or peptide-based PET tracers. Nonetheless, [^89^Zr]Zr-DFO-AP-101 dosimetry (effective dose of 0.21–0.31 mSv/MBq) is lower than or comparable to that of other ^89^Zr-labeled antibody-based radiopharmaceuticals currently undergoing clinical evaluation (e.g., [^89^Zr]Zr-trastuzumab 0.61 mSv/MBq [26], [^89^Zr]Zr-huJ591 0.38 mSv/MBq [37]. In line with our preclinical results, the calculated effective dose of [^89^Zr]Zr-DFO-AP-101 was higher in female participants (0.28 mSv/MBq) than in males (0.21 mSv/MBq). It was found that effective doses in women are generally 20–40% higher than in men, a trend consistently observed across various radiopharmaceuticals due to differences in body mass and volume [38, 39].

The main limitations of this study are related to the small number of participants, particularly the number of ALS patients recruited. This was challenged by the short study duration (12 months of enrolment prior to the end of funding), by the low prevalence of this disease in our geographical region and by the fact that this population have limited mobility and reduced physical capacity to enable them to participate in such a trial (involving up to 5 visits with imaging). Because the number of ALS patients enrolled was very low, 1 enrolled out of 4 planned, we were unable to report robust dosimetry data for this group or perform a meaningful comparison between control volunteers and ALS patients.

In this study, [^89^Zr]Zr-DFO-AP-101 proved to be safe in both control participants and an ALS patient, and the effective radiation dose was within acceptable limits. Further research is required to evaluate the effectiveness of targeted PET imaging to detect mSOD1 in ALS patients or to study its potential for early detection of mSOD1 protein aggregates.

## Supplementary Information

Supplementary Material 1.

## Data Availability

The datasets generated during and/or analysed during the current study are available from the corresponding author on reasonable request.

## Abbreviations

ALS: Amyotrophic lateral sclerosis
CIMS: Sherbrooke molecular imaging center
CT: Computed tomography
DFO: Desferrioxamine
fALS: Familial ALS
GMP: Good manufacturing practices
HPLC: High performance liquid chromatography
MBq/mL: Megabecquerel per milliliter
mSOD1: Misfolded superoxide dismutase-1
NaHCO₃: Sodium bicarbonate
PBS: Phosphate-buffered saline
PET: Positron emission tomography
sALS: Sporadic ALS
SOD1: Superoxide dismutase 1
SUV: Standardized uptake value
VOIs: Volumes of interest
%ID/g: Percentage of the injected dose per gram
