# Supplementary material for: A phase I study to evaluate the dosimetry and safety of [89Zr]Zr-DFO-AP-101, a new antibody-based radiopharmaceutical to detect misfolded SOD1 in amyotrophic lateral sclerosis
Source: Eur J Nucl Med Mol Imaging. 2026 Mar 24;53(7):4639–51. doi: 10.1007/s00259-026-07853-y (PMC13197312; doi:10.1007/s00259-026-07853-y)
Supplement: Supplementary file 1 — Supplementary Material 1. [file 259_2026_7853_MOESM1_ESM.pdf]

A phase I study to evaluate the dosimetry and safety of [^{89}Zr]Zr-DFO-AP-101, a new antibody-based radiopharmaceutical to detect misfolded SOD1 in amyotrophic lateral sclerosis

Etienne Croteau[1], Sébastien Tremblay[2], Jean-François Rousseau[1], Esteban Espinosa-Betancourt[1], Eric Lavallée[1], Stéphanie Dubreuil[1], Samia Ait-Mohand[2], Émilie Lareau-Trudel[3], Sylvie Gosselin[3], Virginie Carrier[4], Sarah Côté-Bigras[4], Etienne Rousseau[1,2,5], Samuel Lemaire-Paquette[4] Marcel Mayer[6], Michael Salzmann[6], Amélie Tétu[4], Marie-Pier Houde[7], Éric Turcotte[1,2,5], Brigitte Guérin[1,2,5].

Supplemental materials

Table of contents

Supplemental Figure 1	S2
Supplemental Figure 2	S2
Supplemental Figure 3	S3
Supplemental Figure 4	S3
Supplemental Figure 5	S4
Quality Control Tests.	S5-S6
Supplemental Tables 1-11	S6-S15

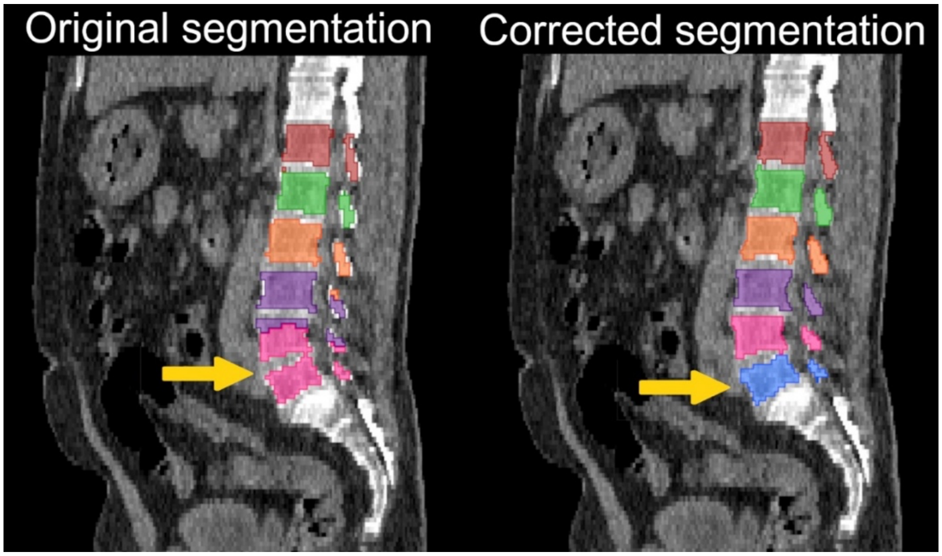

Supplemental Figure 1. Example of manual correction of the segmentation. This participant had an extra lumbar vertebra that was not identified by the spine model.

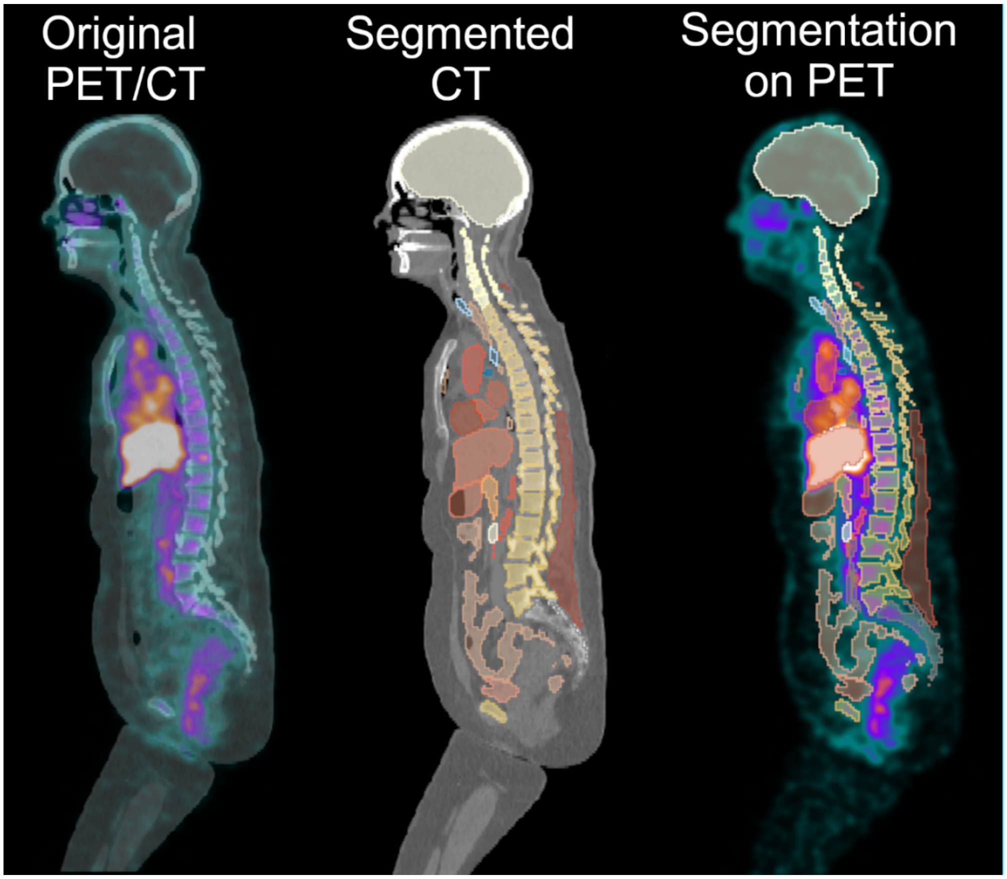

Supplemental Figure 2. Example of image processing. The PET and CT images were registered by the scanner. The CT image was segmented using a pretrained model in MONAI Auto3DSeg. The segmentation was applied to the PET image.

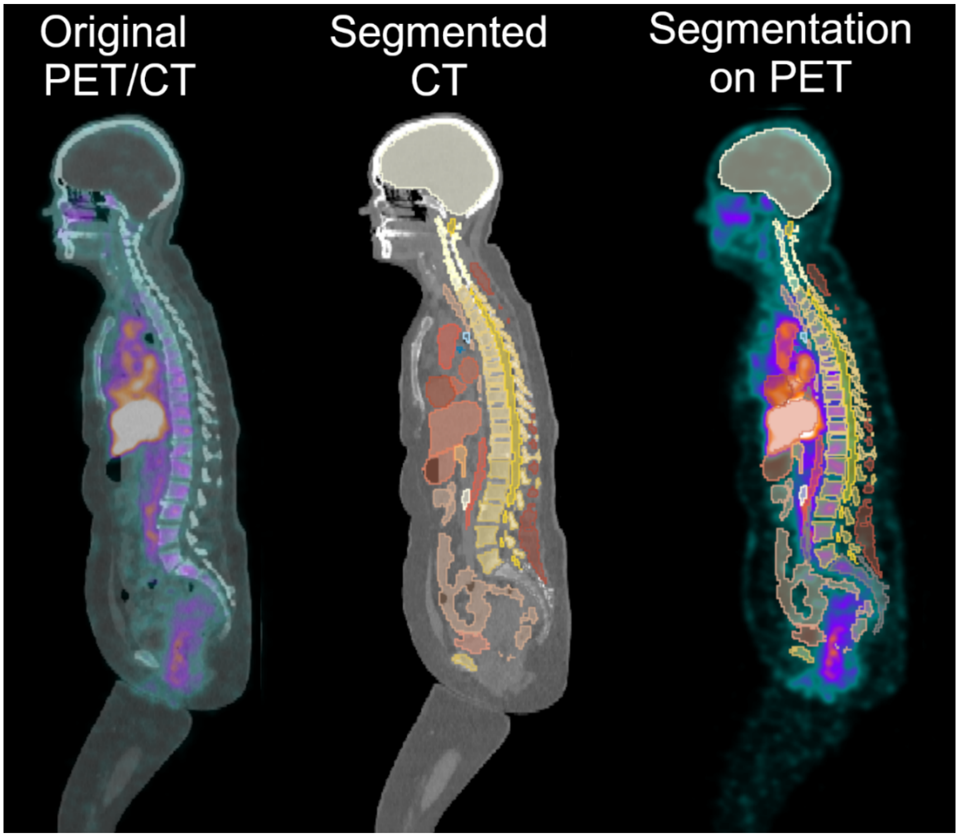

Supplemental Figure 3. Example of the spinal cord segmentation.

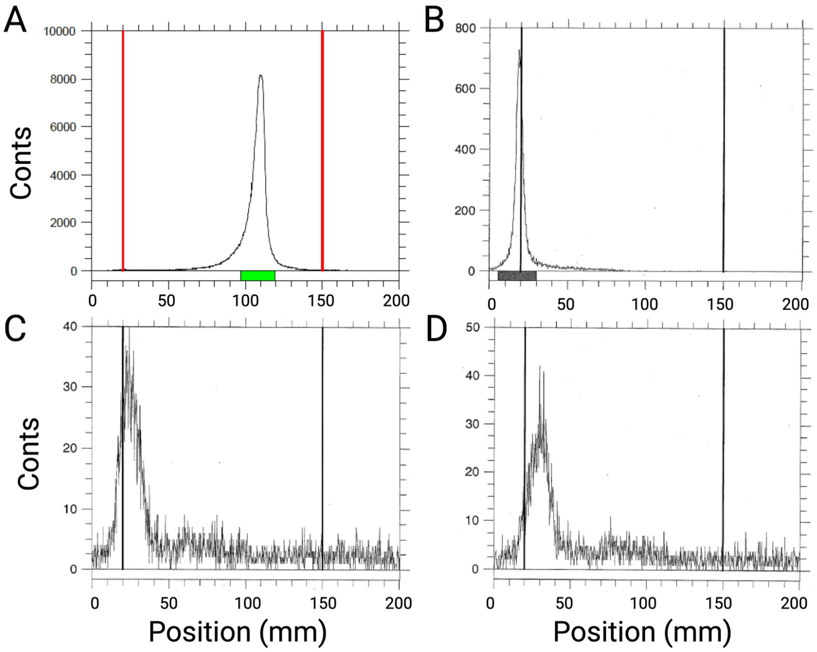

Supplemental Figure 4. Radio-TLC profiles of pure [^{89}Zr]$ZrCl_4$ (A), pure [^{89}Zr]Zr-AP-101 (B) and of [^{89}Zr]Zr-AP-101 in human blood (C) and urine (D) at 2h post-injection.

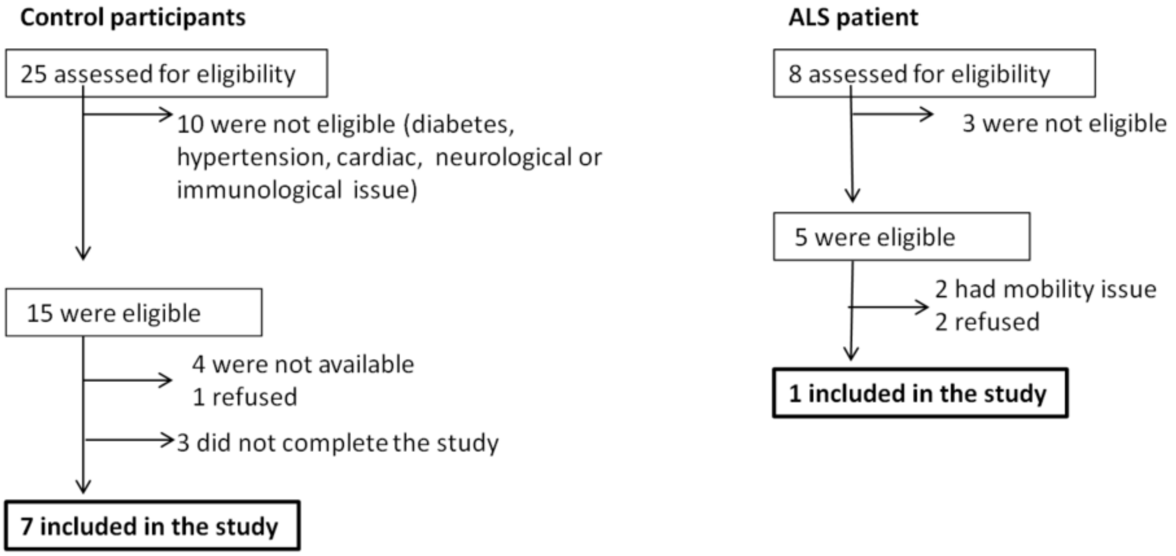

Supplemental Figure 5. Flow diagram

Quality Control Tests. Quality control tests were performed on formulated [^{89}Zr]Zr-DFO-AP-101. The pH was measured with pH strips. High-performance liquid chromatography (HPLC) was used to determine the radiochemical identity and purity. The HPLC system consisted of a quaternary pump (Agilent 1100 Series, model G1311A) connected to a UV detector (Agilent 1100 Series, model G1315B), followed by a flow-through radiometric detector equipped with a photomultiplier tube (Bioscan, series 0605-196). Sample volumes of 15–20 µL were injected using an autosampler (Agilent 1100 Series, model G1329A). Chromatographic separation was performed on an Agilent PRLP-S column (10 µm, 1000 Å, 4.6 × 250 mm) using a gradient elution method. The mobile phase consisted of (A) water with 0.05% formic acid and (B) acetonitrile with 0.05% formic acid. The gradient increased from 5% to 75% B over 10 min., held until 25 min., and then returned to 5% B at 26 min.. Retention times (RT) were compared to a DFO-AP-101 standard for identification. Unlabeled [^{89}Zr]$ZrCl_4$ was analyzed by instant thin-layer chromatography (iTLC) using 50 mM DTPA (pH 4–5) as the mobile phase. Radionuclidic purity was assessed by γ-ray spectrometry using a calibrated high-purity germanium (HPGe) detector with an energy window ranging from 1 to 2,000 keV. Samples were measured for 2 min. immediately after the end of synthesis (EOS) to identify and quantify potential radionuclidic impurities, specifically ^{88}Y (γ peak at 898 keV; half-life: 106.6 days) and ^{88}Zr (γ peak at 393 keV; half-life: 83 days). Gentisic acid content was determined from a 10× stock solution that was diluted into the final formulation prior to filtration. HEPES concentration was quantified as described by Antunes et al., 2020. Briefly, the HPLC system (as previously described) was used with a C18 Phenomenex column (5 µm, 110 Å, 4.6 × 250 mm), operated under isocratic conditions with 20 mM ammonium formate (NH_4HCO_2) at pH 9.5 as the mobile phase. Detection was carried out by UV absorbance. Endotoxin levels were measured using the Limulus Amebocyte Lysate (LAL) assay with the Endosafe-PTS testing system (Charles River Laboratories International). Sterility testing was conducted at CIMS.

Supplemental Table 1. Release specifications for [^{89}Zr]Zr-DFO-AP-101 injectable solution

Test parameter	Test method	Specifications	Validation batches (n=3)	Routine batches (n=7)
Appearance	Visual inspection	Clear, colourless	Conform	Conform
pH	pH strip	4.5-8.0	5.5 ± 0.0	5.1 ± 0.2
Antibody calculation (mg)	Calculation	≤ 10	3.0 ± 0.0	3.0 ± 0.0
Radiochemical identity (%)	HPLC	≤ 10% R.T. of standard	4.7 ± 0.6	4.7 ± 1.9
Radiochemical purity (%)	HPLC	≥ 90	100	99.1 ± 0.8
Radiochemical purity (%)	iTLC	≥ 90	100	100
Radionuclidic purity and identification (%)	Energy spectrum 511KeV & 909 KeV	≥ 98	99.6 ± 0.2	99.8 ± 0.0
Radionuclidic identity (h)	Decay	70.57-86.25	80.2 ± 3.3	78.5 ± 0.9
Radionuclidic impurities (%)	(% of ^{88}Zr and ^{88}Y)	≤ 2	0.1 ± 0.1	0.02 ± 0.04
HEPES buffer	Calculation	≤ 200 µg per injection	Conform	Conform
Gentisic acid calculation	Calculation	≤ 5 mg per injection	Conform	Conform
Filter integrity	Bubble point	Test passed	Conform	Conform
Sterility	Direct inoculation	Sterile	Conform	Conform
Endotoxins	PTS-Endosafe	≤ 175 EU/Volume	Conform	Conform

Supplemental Table 2 | Percentage of injected dose (%ID) in organs, as measured by PET imaging in female participants (n=4) after administration of [^{89}Zr]Zr-DFO-AP-101. The data were not corrected for radioactive decay and correspond to the actual tissue radioactivity burden at each time cspoint.

Percentage of injected dose (mean ± standard deviation)

Organs	Time at Whole body scan				
	2h	D1	D3	D7	D10
Spleen	0.79±0.46	0.62±0.16	0.37±0.14	0.13±0.05	0.07±0.03
Kidney	0.45±0.48	0.82±0.26	0.51±0.18	0.19±0.06	0.10±0.04
Liver	11.88±2.40	11.16±2.98	8.34±1.81	3.99±1.13	2.38±0.55
Stomach	0.78±0.35	0.56±0.22	0.30±0.08	0.11±0.03	0.06±0.02
Lung	2.46±2.18	1.48±1.47	0.80±0.75	0.23±0.22	0.15±0.16
Colon	1.01±0.48	2.16±1.02	0.90±0.64	0.20±0.06	0.13±0.06
Urinary Bladder	3.23±2.14	0.15±0.17	0.06±0.06	0.02±0.02	0.01±0.00
Heart	4.08±2.93	1.85±1.83	1.19±0.66	0.33±0.17	0.18±0.09
Bone	0.34±0.26	0.22±0.15	0.15±0.10	0.07±0.04	0.04±0.03
Brain	0.82±0.08	0.45±0.04	0.24±0.05	0.07±0.01	0.04±0.00
Blood	64.37±7.02	31.79±4.26	14.96±1.62	4.16±0.08	2.14±0.36
Red marrow	1.85±0.76	1.24±0.67	0.66±0.53	0.20±0.23	0.20±0.16

Supplemental Table 3 | Percentage of injected dose (%ID) in organs, as measured by PET imaging in male participants (n=3) after administration of [^{89}Zr]Zr-DFO-AP-101. The data were not corrected for radioactive decay and correspond to the actual tissue radioactivity burden at each time point.

Percentage of injected dose (mean ± standard deviation)					
Organs	Time at Whole body scan				
	2h	D1	D3	D7	D10
Spleen	1.26±0.71	0.62±0.23	0.42±0.21	0.16±0.08	0.09±0.05
Kidney	0.84±0.28	0.78±0.24	0.47±0.22	0.18±0.05	0.11±0.04
Liver	12.82±2.05	10.30±2.61	8.08±1.74	3.96±1.10	2.49±0.47
Stomach	0.56±0.25	0.52±0.18	0.29±0.08	0.12±0.02	0.05±0.01
Lung	3.17±3.11	2.08±2.12	1.19±1.25	0.37±0.29	0.24±0.23
Colon	0.78±0.14	0.81±0.62	0.31±0.18	0.14±0.10	0.07±0.04
Urinary Bladder	4,36±3.11	0.94±1.38	0.15±0.12	0.05±0.06	0.02±0.02
Heart	3.18±2.39	1.89±0.51	0.89±0.44	0.33±0.18	0.16±0.07
Bone	0.98±1.32	0.75±1.03	0.55±0.79	0.26±0.36	0.18±0.25
Brain	0.77±0.12	0.39±0.01	0.20±0.02	0.07±0.01	0.04±0.01
Blood	54.84±5.50	27.65±0.86	11.69±1.03	3.91±1.00	1.95±0.21
Red Marrow	2.12±0.83	1.36±0.71	0.56±0.79	0.31±0.33	0.27±0.23

Supplemental Table 4 | Percentage of injected dose (%ID) in organs, as measured by PET imaging in the female ALS patient (n=1) after administration of [^{89}Zr]Zr-DFO-AP-101. The data were not corrected for radioactive decay and correspond to the actual tissue radioactivity burden at each time point.

Percentage of injected dose (%)					
Organs	Time at Whole body scan				
	2h	D1	D3	D7	D10
Spleen	0.7	0.5	0.4	0.1	0.1%
Kidney	1.1	1.1	0.9	0.4	0.2
Liver	10.7	11.8	10.8	5.2	3.1
Stomach	1.2	0.8	0.3	0.1	0.1
Lung	6.4	3.8	2.2	0.6	0.4
Colon	1.4	1.0	0.5	0.2	0.1
Urinary Bladder	3.5	0.0	0.0%	0.0%	0.0%
Heart	1.3	0.7	0.4	0.1	0.0
Bone	0.2	0.2	0.2	0.1	0.0
Brain	0.7	0.4	0.2	0.1	0.0
Blood	67.7	33.8	14.1	3.4	1.9
Red Marrow	2.9	2.1	1.6	0.7	0.4

Supplemental Table 5. Chemistry and haematology in blood samples, and vital signs of all participants throughout the study

	Mean (± standard deviation)				
Parameters	Screening	Day 1 *	Day 3*	Day 7	Day 10
Sodium (mmol/L)	140.8 (1.04)	141.0 (2.00)	140.5 (1.76)	139.5 (1.51)	139.6 (0.92)
Potassium (mmol/L)	4.19 (0.25)	4.23 (0.30)	4.17 (0.23)	4.08 (0.24)	4.14 (0.21)
Chloride (mmol/L)	105.0 (1.41)	105.2 (2.71)	104.2 (1.84)	103.8 (2.05)	104.6 (1.19)
Calcium (mmol/L)	2.43 (0.07)	2.43 (0.05)	2.41 (0.06)	2.39 (0.04)	2.38 (0.05)
Phosphorus (mmol/L)	0.97 (0.14)	1.09 (0.14)	1.09 (0.13)	1.06 (0.09)	1.05 (0.11)
Magnesium (mmol/L)	0.87 (0.07)	0.88 (0.04)	0.88 (0.05)	0.86 (0.05)	0.88 (0.07)
Glucose (mmol/L)	6.29 (1.09)	5.77 (0.56)	5.55 (0.77)	6.23 (1.12)	6.05 (1.19)
Urea (mmol/L)	6.09 (1.57)	5.70 (1.72)	6.05 (1.76)	5.91 (1.54)	5.73 (1.95)
Total Protein (g/L)	72.45 (2.91)	71.27 (3.93)	72.47 (3.10)	70.55 (3.37)	71.16 (3.45)
Albumin (g/L)	38.84 (2.33)	37.70 (1.08)	38.30 (1.72)	38.21 (2.36)	38.63 (1.82)
Total Bilirubin (µmol/L)	6.24 (2.61)	5.45 (1.77)	5.70 (2.29)	7.10 (3.46)	6.44 (2.57)
ALP (UI/L)	80.75 (23.86)	86.00 (19.48)	83.83 (13.47)	76.63 (24.50)	76.00 (21.88)
AST (UI/L)	29.63 (16.08)	24.50 (5.01)	23.83 (4.67)	26.25 (7.92)	25.50 (8.45)
ALT (UI/L)	32.13 (31.91)	22.33 (10.89)	22.67 (11.04)	30.88 (25.84)	29.63 (25.15)
Creatinine (µmol/L)	73.25 (17.81)	67.17 (11.34)	69.83 (16.65)	74.75 (15.72)	74.63 (16.33)
eGFR (mL/min/1.73m^2)	83.43 (18.18)	89.00 (11.22)	85.67 (14.02)	84.00 (14.10)	85.00 (17.34)
GGT (UI/L)	40.25 (43.35)	25.33 (11.34)	25.00 (10.97)	35.63 (30.97)	35.38 (29.25)
TSH (mUI/L)	2.08 (0.38)	1.85 (0.25)	1.84 (0.41)	2.20 (0.61)	1.90 (0.61)
Hemoglobin (g/L)	140.8 (9.067)	137.3 (4.803)	138.5 (9.006)	137.5 (7.091)	137.4 (9.226)
Hematocrit (%)	41.70 (2.81)	41.00 (1.49)	40.57 (3.07)	40.24 (2.16)	40.26 (2.31)
Erythrocytes (10^{12}/L)	4.62 (0.28)	4.54 (0.27)	4.54 (0.33)	4.49 (0.27)	4.47 (0.27)
Leucocytes (10^9/L)	7.21 (1.49)	7.13 (0.90)	6.83 (1.03)	6.68 (0.95)	6.45 (1.04)
Platelets (10^9/L)	278.0 (56.52)	282.5 (41.89)	287.3 (44.30)	266.0 (44.40)	270.4 (47.58)
Neutrophils (10^9/L)	4.58 (1.53)	4.55 (1.02)	4.20 (1.07)	4.10 (1.16)	3.90 (1.09)
Lymphocytes (10^9/L)	1.83 (0.53)	1.76 (0.34)	1.92 (0.46)	1.77 (0.32)	1.75 (0.47)
Monocytes (10^9/L)	0.51 (0.10)	0.55 (0.17)	0.47 (0.09)	0.52 (0.12)	0.51 (0.11)

Eosinophils (10^9/L)	0.22 (0.09)	0.23 (0.08)	0.20 (0.07)	0.23 (0.09)	0.24 (0.08)
Basophils (10^9/L)	0.07 (0.02)	0.07 (0.03)	0.05 (0.01)	0.05 (0.02)	0.07 (0.02)
Systolic (mmHg)	127.9 (17.27)	123.4 (13.90)	126.3 (8.46)	118.3 (10.98)	122.0 (11.50)
Diastolic (mmHg)	78.88 (5.69)	79.00 (8.35)	78.13 (5.67)	76.25 (5.42)	77.50 (8.33)
Heart rate (beats/min)	75.75 (12.08)	78.75 (13.70)	73.75 (9.19)	78.63 (8.52)	77.13 (11.33)
Temperature (degree Celsius)	36.46 (0.21)	36.43 (0.29)	36.33 (0.35)	36.53 (0.24)	36.30 (0.11)
Oxygen saturation (%)	96.33 (2.08)	95.75 (2.06)	95.50 (1.73)	96.00 (2.00)	97.50 (0.58)

*Data for all 8 patients except for Day 1 and Day 3 where chemistry and haematology were not available for two of them.

Supplemental Table 6 | Percentage of injected dose (%ID) in organs, as measured by PET imaging in female participants (n=4) after administration of [^{89}Zr]Zr-DFO-AP-101. The data were corrected for radioactive decay.

	Percentage of injected dose (mean ± standard deviation)				
Organs	Time at whole body scan				
	2h	D1	D3	D7	D10
Spleen	0.99±0.46	0.76±0.20	0.68±0.26	0.55±0.23	0.53±0.29
Kidney	0.81±0.49	1.02±0.32	0.94±0.32	0.84±0.28	0.77±0.22
Liver	12.09±2.44	13.74±3.64	15.56±3.54	17.41±4.84	18.94±5.81
Stomach	0.80±0.36	0.69±0.28	0.56±0.15	0.48±0.12	0.50±0.17
Lung	2.50±1.83	1.83±1.83	1.47±1.35	1.03±0.98	1.09±0.95
Colon	1.03±0.49	2.66±1.27	1.68±1.20	0.85±0.27	1.00±0.32
Urinary Bladder	3.29±2.18	0.19±0.22	0.12±0.12	0.09±0.09	0.07±0.03
Heart	3.84±3.41	2.27±2.24	2.23±1.23	1.16±0.75	1.44±0.79
Bone	0.18±0.10	0.28±0.19	0.28±0.18	0.29±0.20	0.30±0.17
Brain	0.84±0.08	0.56±0.05	0.44±0.08	0.30±0.03	0.31±0.06
Blood	65.52±7.14	39.16±5.24	27.82±2.60	18.20±0.33	16.56±2.18
Red marrow	1.63±0.80	1.30±0.82	1.00±0.88	0.70±0.89	1.12±1.19

Supplemental Table 7 | Percentage of injected dose (%ID) in organs, as measured by PET imaging in male participants (n=3) after administration of [^{89}Zr]Zr-DFO-AP-101. The data were corrected for radioactive decay.

	Percentage of injected dose (mean ± standard deviation)				
Organs	Time at whole body scan				
	2h	D1	D3	D7	D10
Spleen	1.29±0.73	0.76±0.28	0.77±0.34	0.67±0.38	0.61±0.33
Kidney	0.86±0.28	0.97±0.29	0.86±0.37	0.75±0.26	0.75±0.23
Liver	13.05±2.09	12.77±3.27	14.98±3.70	16.03±3.03	16.56±3.36
Stomach	0.57±0.26	0.64±0.23	0.54±0.13	0.49±0.14	0.36±0.05
Lung	3.22±3.16	2.57±2.62	2.14±2.16	1.57±1.30	1.59±1.46
Colon	0.79±0.14	1.00±0.77	0.58±0.35	0.55±0.35	0.45±0.30
Urinary Bladder	4,44±3.17	1.17±1.71	0.27±0.22	0.16±0.21	0.11±0.14
Heart	3.24±2.43	2.34±0.64	1.66±0.86	1.27±0.60	1.06±0.45
Bone	1.00±1.34	0.92±1.27	0.98±1.38	1.11±1.61	1.17±1.64
Brain	0.78±0.12	0.48±0.02	0.36±0.03	0.28±0.06	0.24±0.05
Blood	52.82±5.60	34.24±0.94	21.52±1.05	15.33±1.20	12.90±136
Red Marrow	2.16±0.85	1.68±0.87	1.00±1.38	1.40±1.43	1.76±1.51

Supplemental Table 8 | Percentage of injected dose (%ID) in organs, as measured by PET imaging in the female ALS patient (n=1) after administration of [^{89}Zr]Zr-DFO-AP-101. The data were corrected for radioactive decay.

	Percentage of injected dose (%)				
Organs	Time at whole body scan				
	2h	D1	D3	D7	D10
Spleen	0.7	0.6	0.7	0.6	0.6
Kidney	1.1	1.4	1.6	1.7	1.4
Liver	10.9	14.5	20.0	22.6	25.4
Stomach	1.2	0.9	0.6	0.6	0.8
Lung	6.5	4.6	4.0	2.7	2.9
Colon	1.4	1.2	0.8	0.7	0.8
Urinary Bladder	3.6	0.0	0.0	0.1	0.0
Heart	5.1	3.2	2.7	1.4	1.5
Bone	0.2	0.2	0.3	0.3	0.3
Brain	0.7	0.4	0.4	0.3	0.3
Blood	68.3	41.5	26.0	14.8	15.4
Red Marrow	3.0	2.6	3.0	2.9	3.6

Supplemental Table 9 | Percentage of injected dose per gram (%ID/g) of tissue, as measured by PET imaging in female participants (n=4) after administration of [^{89}Zr]Zr-DFO-AP-101. The data were corrected for radioactive decay.

Organs	Percentage of injected dose/g of tissue (mean ± standard deviation)				
	Time at whole body scan				
	2h	D1	D3	D7	D10
Lumbar vertebrae	0.0023±0.0004	0.0021±0.0003	0.0020±0.0002	0.0020±0.0003	0.0023±0.0004
Thoracic vertebrae	0.0028±0.0003	0.0022±0.0004	0.0022±0.0003	0.0021±0.0002	0.0023±0.0004
Spinal cord	0.0017±0.0002	0.0014±0.0003	0.0013±0.0003	0.0012±0.0001	0.0013±0.0001
Femoral head	0.0006±0.0003	0.0006±0.0004	0.0006±0.0003	0.0007±0.0003	0.0007±0.0003

Supplemental Table 10 | Percentage of injected dose per gram (%ID/g) of tissue, as measured by PET imaging in male participants (n=3) after administration of [^{89}Zr]Zr-DFO-AP-101. The data were corrected for radioactive decay.

Organs	Percentage of injected dose/g of tissue (mean ± standard deviation)				
	Time at whole body scan				
	2h	D1	D3	D7	D10
Lumbar vertebrae	0.0022±0.0001	0.0018±0.0003	0.0018±0.0004	0.0019±0.0006	0.0019±0.0007
Thoracic vertebrae	0.0023±0.0002	0.0018±0.0002	0.0018±0.0003	0.0019±0.0005	0.0020±0.0006
Spinal cord	0.0013±0.0001	0.0010±0.0002	0.0010±0.0002	0.0010±0.0003	0.0011±0.0004
Femoral head	0.0003±0.0000	0.0003±0.0000	0.0003±0.0001	0.0004±0.0001	0.0004±0.0001

Supplemental Table 11 | Percentage of injected dose per gram (%ID/g) of tissue, as measured by PET imaging in the female ALS patient (n=1) after administration of [^{89}Zr]Zr-DFO-AP-101. The data were corrected for radioactive decay.

Organs	Percentage of injected dose (%)/g of tissue				
	Time at whole body scan				
	2h	D1	D3	D7	D10
Lumbar vertebrae	0.0023	0.0020	0.0023	0.0024	0.0029
Thoracic vertebrae	0.0026	0.0022	0.0025	0.0024	0.0029
Spinal cord	0.0019	0.0015	0.0014	0.0014	0.0017
Femoral head	0.0005	0.0006	0.0005	0.0005	0.0006
